# Silicon attenuates nutritional disorder of phosphorus in seedlings of *Eucalyptus grandis* × *Eucalyptus urophylla*

**DOI:** 10.1186/s12870-024-05147-9

**Published:** 2024-05-29

**Authors:** Eduarda Gonçalves Reis, Rinaldo Cesar de Paula, Jonas Pereira de Souza Júnior, Renato de Mello Prado, Mariana Bomfim Soares, Kleve Freddy Ferreira Canteral

**Affiliations:** 1 School of Agricultural and Veterinarian Sciences, Department of Agricultural Sciences, São Paulo State University (UNESP), Via de acesso Prof. Paulo Donato Castellane s/n., Jaboticabal, São Paulo, 14884900 Brazil; 2 Citrus Research and Education Center, Universtiy of Florida, 700 experiment Station Rd, Lake Alfred, FL 33850 USA

**Keywords:** Beneficial element, Macronutrient, Nutritional deficiency, Nutritional toxicity

## Abstract

**Background:**

Nutritional disorders of phosphorus (P), due to deficiency or toxicity, reduce the development of *Eucalyptus* spp. seedlings. Phosphorus deficiency often results in stunted growth and reduced vigor, while phosphorus toxicity can lead to nutrient imbalances and decreased physiological function. These sensitivities highlight the need for precise management of P levels in cultivation practices. The use of the beneficial element silicon (Si) has shown promising results under nutritional stress; nevertheless, comprehensive studies on its effects on *Eucalyptus* spp. seedlings are still emerging. To further elucidate the role of Si under varying P conditions, an experiment was conducted with clonal seedlings of a hybrid *Eucalyptus* spp. (*Eucalyptus grandis *× *Eucalyptus urophylla*, A207) in a soilless cultivation system. Seedlings were propagated using the minicutting method in vermiculite-filled tubes, followed by treatment with a nutrient solution at three P concentrations: a deficient dose (0.1 mM), an adequate dose (1.0 mM) and an excessive dose (10 mM), with and without the addition of Si (2mM). This study assessed P and Si concentration, nutritional efficiency, oxidative metabolism, photosynthetic parameters, and dry matter production.

**Results:**

Si supply increased phenolic compounds production and reduced electrolyte leakage in seedlings provided with 0.1 mM of P. On the other hand, Si favored quantum efficiency of photosystem II as well as chlorophyll *a* content in seedlings supplemented with 10 mM of P. In general, Si attenuates P nutritional disorder by reducing the oxidative stress, favoring the non-enzymatic antioxidant system and photosynthetic parameters in seedlings of *Eucalyptus grandis *× *Eucalyptus urophylla*.

**Conclusion:**

The results of this study indicate that *Eucalyptus grandis *× *Eucalyptus urophylla* seedlings are sensitive to P deficiency and toxicity and Si has shown a beneficial effect, attenuating P nutritional disorder by reducing the oxidative stress, favoring the non-enzymatic antioxidant system and photosynthetic parameters.

## Introduction

The rising global demand for wood and its derivatives has catalyzed the expansion of commercial forest areas [1], leading to increased production of seedlings, particularly among forest species such as the *Eucalyptus* [2, 3]. Native to Australia, *Eucalyptus* trees are celebrated for their multifaceted utility in both medicinal and industrial domains. The extraction of eucalyptus essential oil from the tree leaves, renowned for its antimicrobial, anti-inflammatory, and antiseptic properties, serves pivotal roles in the pharmaceutical, cosmetics, and food industries. This essential oil is predominantly composed of 1,8-cineole (eucalyptol), a compound whose antimicrobial and anti-inflammatory activities have been well-documented, affirming its extensive application across these sectors [4]. Industrially, *Eucalyptus* wood is a core material in the pulp industry for paper production and is increasingly utilized in the manufacture of cross-laminated timber (CLT). The strength and mechanical properties of *Eucalyptus* CLT have proven comparable to those of traditional softwoods, making it a viable alternative for structural applications [5].

The *Eucalyptus grandis* × *Eucalyptus urophylla* hybrid, where *Eucalyptus grandis* is the female progenitor and *Eucalyptus urophylla* is the male progenitor, is economically significant for energy production, cellulose production, and forest preservation [6]. The genome of this hybrid has been sequenced, revealing a recent whole-genome duplication event and expansions in gene families related to various metabolic pathways and plant-pathogen interactions [7]. Several factors influence the success of forest crops, such as water and nutrient availability [8, 9]. Phosphorus (P) is an important nutrient for eucalypt plantation and plays a crucial role in the growth of *Eucalyptus* spp. seedlings as well as in their initial establishment in the field, favoring earlier harvesting and increasing crop yield [8–11]. Thus, P is vital for plant growth and its availability in the soil affects its uptake, transport, and utilization, ultimately influencing nutritional efficiency [5].

Success in the establishment stage of a perennial crop is highly dependent on nutrition, mainly phosphate, which has a direct impact on the quality of seedlings [12]. Thereby, P nutritional disorder can compromise seedling development [13, 14]. Studies commonly investigate P deficiency, since it causes a change in the photosynthetic apparatus and an increased production of reactive oxygen species in chloroplasts, leading to a reduction in the concentration of photosynthetic pigments [15–17]. However, the negative effects of P excess are poorly studied for *Eucalyptus* spp. seedlings.

The use of beneficial elements, such as silicon (Si), has shown promising results under P nutritional disorder [18]; nevertheless, most studies focus on Si-accumulating plants, requiring further investigations with other plant groups [19]. The genus *Eucalyptus* belongs to the Myrtaceae family and is classified as a non-Si-accumulating plant [20] However, a recent study indicated that Si attenuates the harmful effects of abiotic stress, such as ammonium toxicity on *Eucalyptus* spp. seedlings, by increasing the concentration of photosynthetic pigments and quantum efficiency of photosystem II [21]. In addition, Si is reported to attenuate P deficiency and toxicity in some species [22–24], but studies on *Eucalyptus* spp. seedlings are scarce.

Therefore, in this study, we aimed to test the hypotheses: (i) new hybrids of *Eucalyptus* spp. is sensitive to P deficiency and toxicity. If so, (ii) Si supply can attenuate the negative effect of P nutritional disorder through physiological and nutritional mechanisms by reducing oxidative stress, while increasing photosynthetic parameters and nutritional efficiencies. Here, we assessed the effects of Si supply on seedlings of *Eucalyptus grandis *× *Eucalyptus urophylla* grown underdosage, adequate dosage, and overdose of P.

Given the expansion of *Eucalyptus* spp. cultivation worldwide to in marginal areas in weathered soils, soils with high and low P adsorption are common, which increase the risk of P deficiency and toxicity in plants, respectively. Therefore, if the hypotheses studied are accepted and the use of Si, an environmentally friendly element, is capable of mitigating nutritional disorders of P, while strengthening the sustainability of *Eucalyptus* spp. cultivation, its use becomes an alternative aligned with the global agenda of Sustainable Development Goals.

## Methods

### Seedling propagation and growth conditions

An experiment was conducted with clonal seedlings of a hybrid of *Eucalyptus grandis *× *Eucalyptus urophylla* hybrid, provided by ArboGen, in a soilless cultivation system, from March to June 2021 in a greenhouse at the School of Agricultural and Veterinary Sciences of the Sao Paulo State University, Campus Jaboticabal, Brazil.

The cloned seedlings were propagated on March 13, 2021, using the minicutting method, described by Xavier [25]. These methods involved placing mini-cuttings with 3 to 5 cm in length with a pair of leaves from the apex of the branches into plastic tuber of 55 cm³, filled with fine-grained vermiculite (diameter < 0.1 mm). The cuttings were treated with 1,000 mg L^− 1^ of indolebutyric acid (IBA) in powder form, and them placed in a greenhouse environment maintained at 27 ± 2 °C with relative humidity above 80%. After 30 days, the rooted cuttings underwent various treatments.

After the rooting of minicuttings, the seedlings received 5 mL of water for 20 days. Next, with the seedlings measuring 55 cm in height, the application of 10 mL daily nutrient solution -P [26] was started with modification of the Fe source to Fe-EDDHMA, and the pH adjusted to 5.0 ± 0.5 with the use of NaOH (1 M) or HCl (1 M) solution. Initially, the nutrient solution was applied for a 7-day period, at 25% of the concentration indicated by Hoagland and Arnon [26]. Afterward, the solution concentration was increased to 50% for one week and then to 75%, which was kept until the end of the experimental period.

Drainage was performed once a day throughout the entire experimental period to prevent substrate salinization. For this purpose, 5 mL of deionized water was added to the substrate to induce drainage of the nutrient solution, which was discarded. After 12 h, a new nutrient solution was added to the tubes and the nutrients necessary for seedling development were supplied during the rest of the plant cycle.

### Experimental design and treatments

The experiment was developed in a completely randomized design in a 3 × 2 factorial scheme, with three concentrations of P: 0.1 mM under dose, 0,1 mM (under dose), 1,0 mM (adequate dose), and 10 mM (overdose) in the absence and presence (2 mmol L^− 1^) of Si. Phosphorus concentrations were based on the recommendation of Hoagland and Arnon [26] corresponding to 10, 100, and 1000% of the recommended value, respectively. Potassium phosphate (KH_2_PO_4_) was used as a P source, and the K content was balanced across treatments by adding potassium chloride. The Si concentration was based on preliminary studies to avoid Si polymerization in the solution. Sodium silicate (Na_2_SiO_3_) was used as a Si source and the Na content was balanced between treatments through the addition of sodium chloride.

The treatments (P and Si) were added to the nutrient solution and 10 mL of the nutrient solution -P was consistently applied every day throughout the entire experimental period.

### Oxidative metabolisms and non-enzymatic defense mechanisms

At 95 days after the start of treatments, oxidative stress was assessed based on electrolyte leakage. For this analysis, five leaf discs were collected with an area of ​​129 mm² each from the second fully developed leaf. The leaf discs were placed in a beaker with 20 mL of deionized water for 2 h at room temperature (25 ± 2 °C). After this period, electrical conductivity (EC) of the solution was measured (EC1), using a bench conductivity meter. Subsequently, the samples were placed in an autoclave at 120 °C for 20 min, followed by a new measurement of electrical conductivity (EC2). Electrolyte leakage was then calculated as:

$$Electrolyte\,leakage{\text{}} = \frac{{EC1}}{{EC2}} \times100$$

The content of total phenolic compounds and carotenoids were determined as non-enzymatic antioxidant components. The content of total phenols was determined according to the methodology adapted by Singleton and Rossi [27]. For this, discs with 0.05 g of fresh mass were collected from the leaves and transferred to 15 mL falcon tubes wrapped in aluminum foil. Concentrated methanol (2 mL) was added to the tubes, which were capped and placed in a water bath at 25 °C for 3 h. Then, the discs were removed, and the aliquot filtered, followed by the addition of 3 mL of methanol. For the colorimetric reaction, 1 mL of the filtered extract was transferred to another 15 mL falcon tube wrapped in aluminum foil, to which 15 mL of distilled water and 0.5 mL of 2 N Folin-Ciocalteu reagent were added and kept for 3 min. Next, 1.5 mg of sodium carbonate was added and kept for another 2 h. After completion, the absorbance was read at a wavelength of 765 nm using a spectrophotometer.

The carotenoid content was determined according to Lichtenthaler [28]. For this, discs with 0.05 g of fresh leaves were collected, kept in 80% ketone under refrigeration and in the dark for depigmentation and read on a spectrophotometer.

### Photosynthetic pigments and quantum efficiency of photosystem II

At 95 days after the start of treatments, measurements of chlorophyll *a* and *b* contents and quantum efficiency of photosystem II were carried out during the same period of the assessments of non-enzymatic oxidative metabolism.

The chlorophyll *a* and *b* contents were determined according to Lichtenthaler [28], in a process similar to that used to determine the content of carotenoids, with ketone depigmentation and reading on a spectrophotometer.

Quantum efficiency of photosystem II (Fv/Fm) was measured using a fluorometer (OS-30p, Opti-Sciences) in evaluations carried out on the second fully developed leaf, from 7h00 to 8h00. For reading, tweezers were attached to the leaves, which were kept in the dark for 30 min to stabilize photosynthetic reaction rates. Subsequently, measurements were taken based on the reading with the fluorometer.

### Production of shoot, root, and total dry mass

At 100 days after the initiation of treatments, which marked the end of the experiment, the seedlings were collected, segmented into shoots and roots, and then decontaminated by washing with water, detergent solution (0.1%), acid solution (HCl – 0.3%), and deionized water. After decontamination, the samples were dried in a forced air circulation oven at 65 ± 5 °C until a constant mass was obtained to determine the dry mass (DM). Afterward, the material was weighed to quantify DM production of shoots (SDM) and roots (RDM). The total DM production and the rate between RDM and SDM were also obtained, calculated by the sum and the quotient, respectively, between production RDM and SDM.

### Nutritional assessments of P and Si

The P content was determined from acidic digestion of the ground samples, followed by a colorimetric reaction with ammonium molybdate and sodium vanadate and then by reading on a spectrophotometer [29]. The Si content was determined from the alkaline digestion of 0.1 g of DM of plant material with H_2_O_2_ and NaOH in an oven at 90 °C for 4 h [30], followed by a colorimetric reaction with ammonium molybdate in an acidic medium (oxalic acid and hydrochloric acid) and the Si concentration was determined by reading on a spectrophotometer [31]. the P and Si accumulation was obtained by multiplying the P and Si content by RDM, SDM and total DM production.

Absorption, transport, and utilization efficiencies of P were also calculated. Absorption efficiency was calculated considering the ratio between total P accumulation and RDM, as described by Swiader [32]:

$$Absorption\,efficiency\left( {g{g^{ - 1}}} \right) = \frac{{P total\,accumulation}}{{Root\,dry\,mass}}$$

Transport efficiency was calculated considering the ratio between P accumulation in shoots and the RDM using the formula described by Li [33]:

$$Transport\,efficiency\left( \% \right) = \frac{{P\,accumulation\,in\,shoots}}{{Root\,dry\,mass}} \times 100$$

Utilization efficiency was calculated considering the ratio between squared total dry mass and total P accumulation in the seedling using the formula described by Siddiqi and Glass [34]:

$$Utilization\,efficiency\left( {{g^2}\,{g^{ - 1}}} \right) = \frac{{Total\,Dry\,Mass\,{P^2}}}{{Total\,accumulation\,of\,P}}$$

### Statistical analysis

The data obtained were preliminarily analyzed for homoscedasticity and then subjected to the analysis of variance (F-Test). When significant, the means were compared using the Tukey test at 5% probability. The analyses were carried out using Sisvar® Software [35]. The results were presented in figures to highlight significant interactions between phosphorus treatment and silicon supply. In cases where no interaction was detected, the figures illustrated the isolated effects of phosphorus concentrations and silicon supply separately.

In addition, the hierarchical cluster analysis (HCA) was carried out, based on the Euclidean distance using the similarity coefficient, the single linkage method as a group connection algorithm, and the principal component analysis (PCA), based on the data correlation. The HCA and PCA were performed using the Python programming language (3.9.7; Python Software Foundation).

## Results

### Phosphorus and silicon concentration and accumulation in shoots and roots

**Fig. 1:**
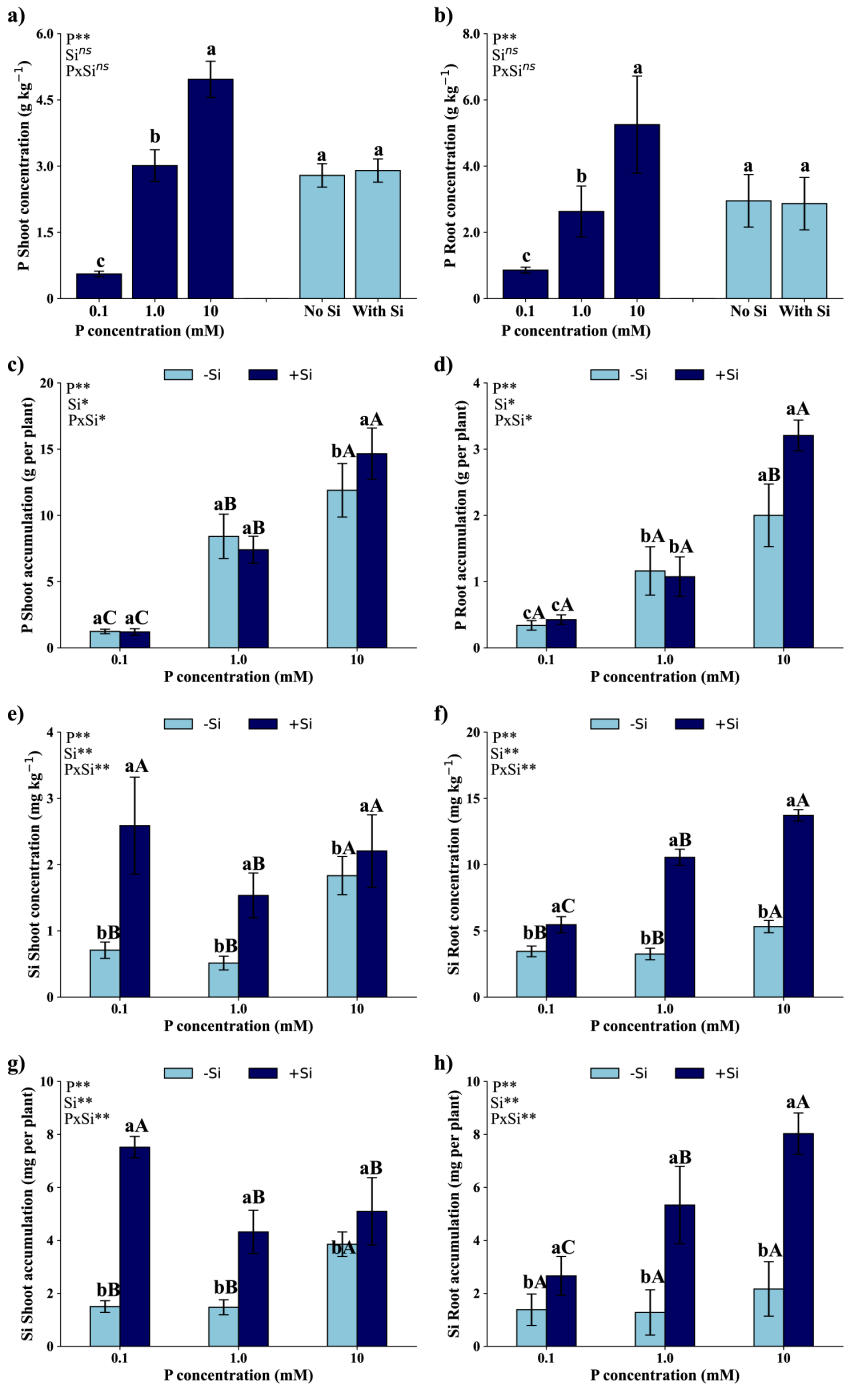
Phosphorus (P) concentration in the shoot (**a**) and roots (**b**), P accumulation in the shoot (**c**) and roots (**d**), silicon (Si) concentration in the shoot (**e**) and roots (**f**), and Si accumulation in the shoot (g) and roots (h) of *Eucalyptus grandis* × *Eucalyptus urophylla* seedlings cultivated under different P concentrations (0.1, 1.0, and 10 mM) in the absence (-Si) and presence (+ Si) of Si. **, * and ^ns^ – significant at 1 and 5% probability levels and non-significant by the F test. Lowercase letters compare the isolated effects of P or Si in the presence and absence of Si at the same P concentration. Uppercase letters compare different P concentrations under the same Si condition

The interaction between P dosages and application or not of Si was not significant (*p* > 0.05) on the P concentration in shoots (Fig. 1a) and roots (Fig. 1b). However, there was a significant interaction (*p* < 0.05) between these factors in P accumulation, both in shoots (Fig. 1c) and roots (Fig. 1d). P concentrations were higher in shoots and roots in treatments with 10 mM of P, followed by treatments with 1.0 and 0.1 mM of P, respectively. Differences regarding the application or not of Si were not significant (*p* > 0.05) regarding P concentration in shoots (Fig. 1a) and roots (Fig. 1b).

P accumulation in shoots (Fig. 1c) and roots (Fig. 1d), in the presence or absence of Si, was more pronounced in seedlings grown with 10 mM, followed by treatments with 1.0 mM and 0.1 mM. There were no significant differences between the presence and absence of Si in treatments with 1.0 mM and 0.1 mM of P. However, in the treatment with 10 mM, greater accumulation of P was observed in seedlings grown in the presence of Si.

A significant interaction (*p* < 0.01) was observed between P dosages and application or not of Si on the Si concentration in both shoots (Fig. 1e) and roots (Fig. 1f), as well as Si accumulation in shoots (Fig. 1g) and roots (Fig. 1h). Without Si application, Si concentration in shoots was higher when the seedlings were grown with 10mM of P, without significant differences between dosages of 0.1 and 1.0 mM of P. On the other hand, with Si application, the adoption of 0.1 mM and 10 mM of P increased Si concentration in shoots, compared to the application of 1.0 mM of P (Fig. 1e).

Si concentration in roots (Fig. 1f), in the absence of Si, showed a similar pattern to the Si concentration in shoots (Fig. 1e) and was higher in seedlings that received 10 mM of P, without significant differences between the concentrations of 0.1 mM and 1.0 mM of P. In the presence of Si, Si concentration in roots increased as the applied dosage of P increased.

Si accumulation in shoots (Fig. 1g), in the absence of Si, was greater in seedlings grown with 10 mM of P, with no difference between the concentrations in treatments with 0.1 and 1.0 mM of P. In the presence of Si, however, greater Si accumulation was observed in shoots of seedlings that received 0.1 mM of P, with no significant difference between treatments with 1.0 mM and 10 mM of P.

Si accumulation in roots (Fig. 1h), in the absence of Si, did not present significant variations depending on the dosage of P studied. However, in the presence of Si, Si accumulation in roots increased with the increasing P concentrations applied.

Si concentration in shoots (Fig. 1e) and roots (Fig. 1f) and Si accumulation in shoots (Fig. 1g) and roots (Fig. 1h) were higher in plants that received Si compared to seedlings grown in the absence of Si, regardless of the P dosage studied.

### Nutritional efficiencies

**Fig. 2:**
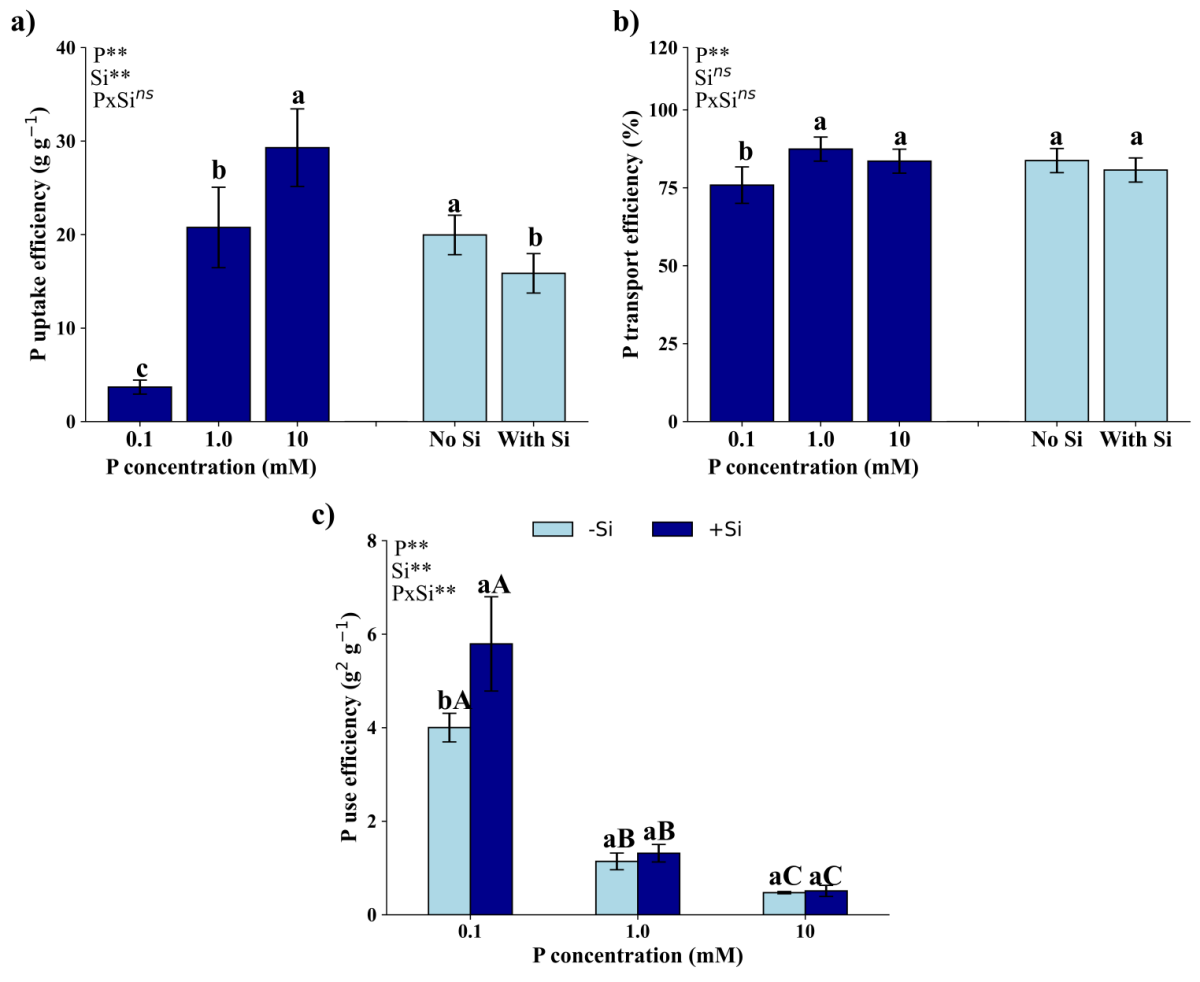
Phosphorus (P) absorption efficiency (**a**), transportation efficiency (**b**), and utilization efficiency (**c**) of *Eucalyptus grandis* × *Eucalyptus urophylla* seedlings cultivated under different P concentrations (0.1, 1.0, and 10 mM) in the absence (-Si) and presence (+ Si) of silicon. **, * and ^ns^ – significant at 1 and 5% probability levels and non-significant by the F test. Lowercase letters indicate differences in P concentrations, and uppercase letters indicate the presence or absence of Si, according to the Tukey test at 5% probability

The interaction between P dosages and application or not of Si was not significant (*p* > 0.05) regarding P absorption (Fig. 2a) and transport (Fig. 2b) efficiencies; however, it was significant (*p* < 0.05) regarding P use efficiency (Fig. 2c).

Absorption efficiency of P by *Eucalyptus grandis *× *Eucalyptus urophylla* was higher by seedlings cultivated with 10 mM of P, followed by treatments with 1.0 mM of P and with 0.1 mM of P (Fig. 2a). Additionally, P absorption efficiency was higher in seedlings without application of Si, compared to seedlings that received Si application.

Transport efficiency was higher in seedlings grown under concentrations of 10 mM and 1.0 mM of P, compared to seedlings grown under 0.1 mM of P. Si did not show any effects on P transport efficiency (Fig. 2b).

Use efficiency of P was higher in seedlings that received 0.1 mM of P, followed by those that received 1.0 mM, and then 10 mM of the nutrient. Silicon increased the use efficiency of P only in plants that received 0.1 mM of P, with no difference observed in the other studied P dosages.

### Oxidative metabolism and non-enzymatic mechanisms defense

**Fig. 3:**
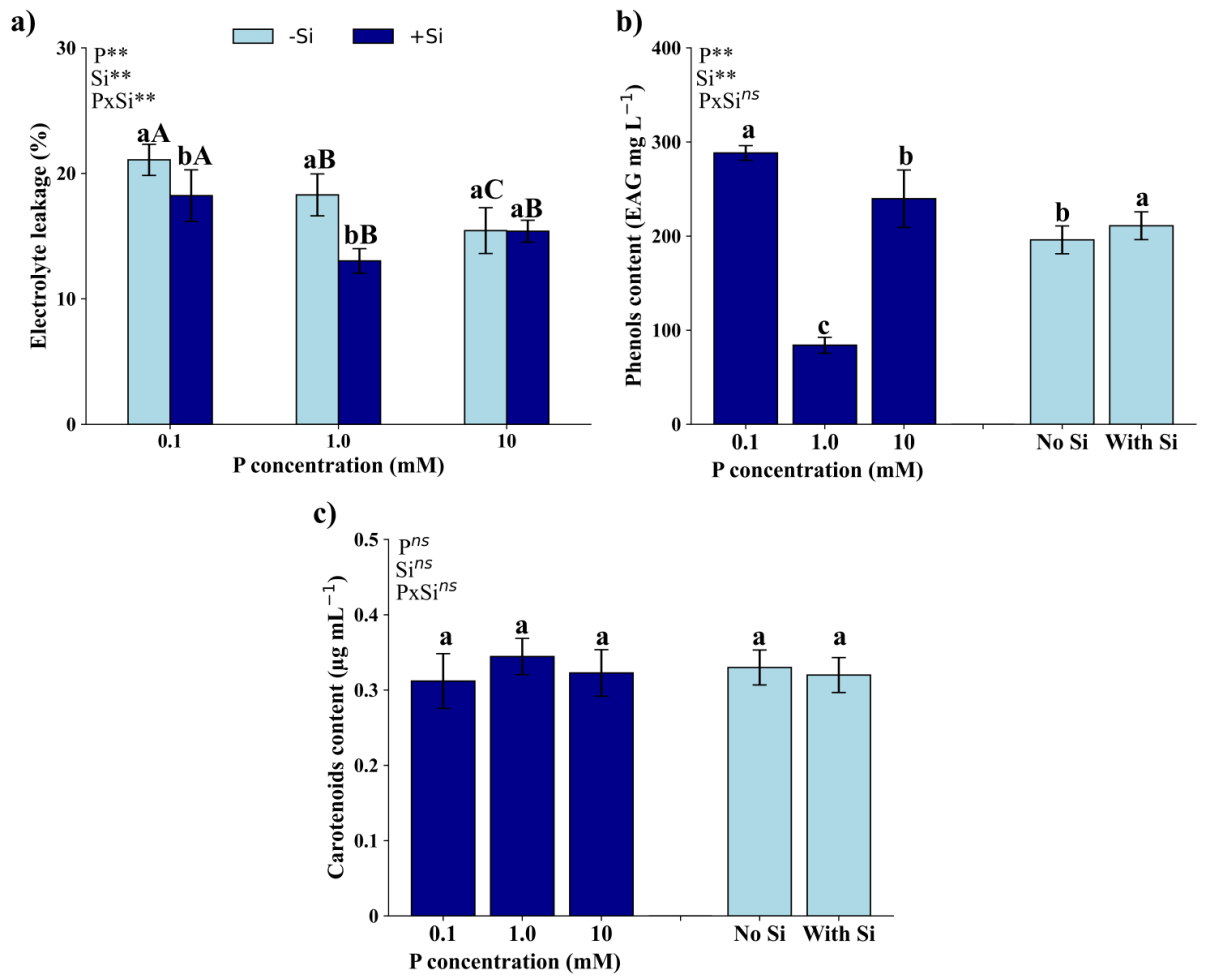
Electrolyte leakage (**a**), total phenolic concentration (**b**), and carotenoid concentration (**c**) of *Eucalyptus grandis* × *Eucalyptus urophylla* seedlings cultivated under different P concentrations (0.1, 1.0, and 10 mM) in the absence (-Si) and presence (+ Si) of silicon. **, * and ^ns^ – significant at 1 and 5% probability levels and non-significant by the F test. Lowercase letters compare the isolated effects of P or Si in the presence and absence of Si at the same P concentration. Uppercase letters compare different P concentrations under the same Si condition

There was a significant interaction (*p* < 0.01) between P and Si dosages on electrolyte leakage (Fig. 3a). However, the interaction between these factors was not significant (*p* > 0.05) on the content of total phenols (Fig. 3b) and carotenoids (Fig. 3c). For the content of carotenoids, the isolated effect of P and Si was also not significant.

Electrolyte leakage (Fig. 3a), in the absence of Si, decreased with increasing P dosage applied to the seedlings. In the presence of Si, however, electrolyte leakage was more pronounced in seedlings grown under 0.1 mM of P, with no significant differences for seedlings grown under 1.0 and 10 mM of P. At dosages of 0.1 and 1.0 mM of P, electrolyte leakage was more significant in seedlings grown in the absence of Si, while at a dosage of 10 mM of P, no significant differences were observed due to the presence of Si.

The concentration of total phenols (Fig. 3b) was higher in seedlings that received 0.1 mM of P, followed by those with 10 mM of P and by those that received 1.0 mM of P.

### Photosynthetic pigments and quantum efficiency of photosystem II

**Fig. 4:**
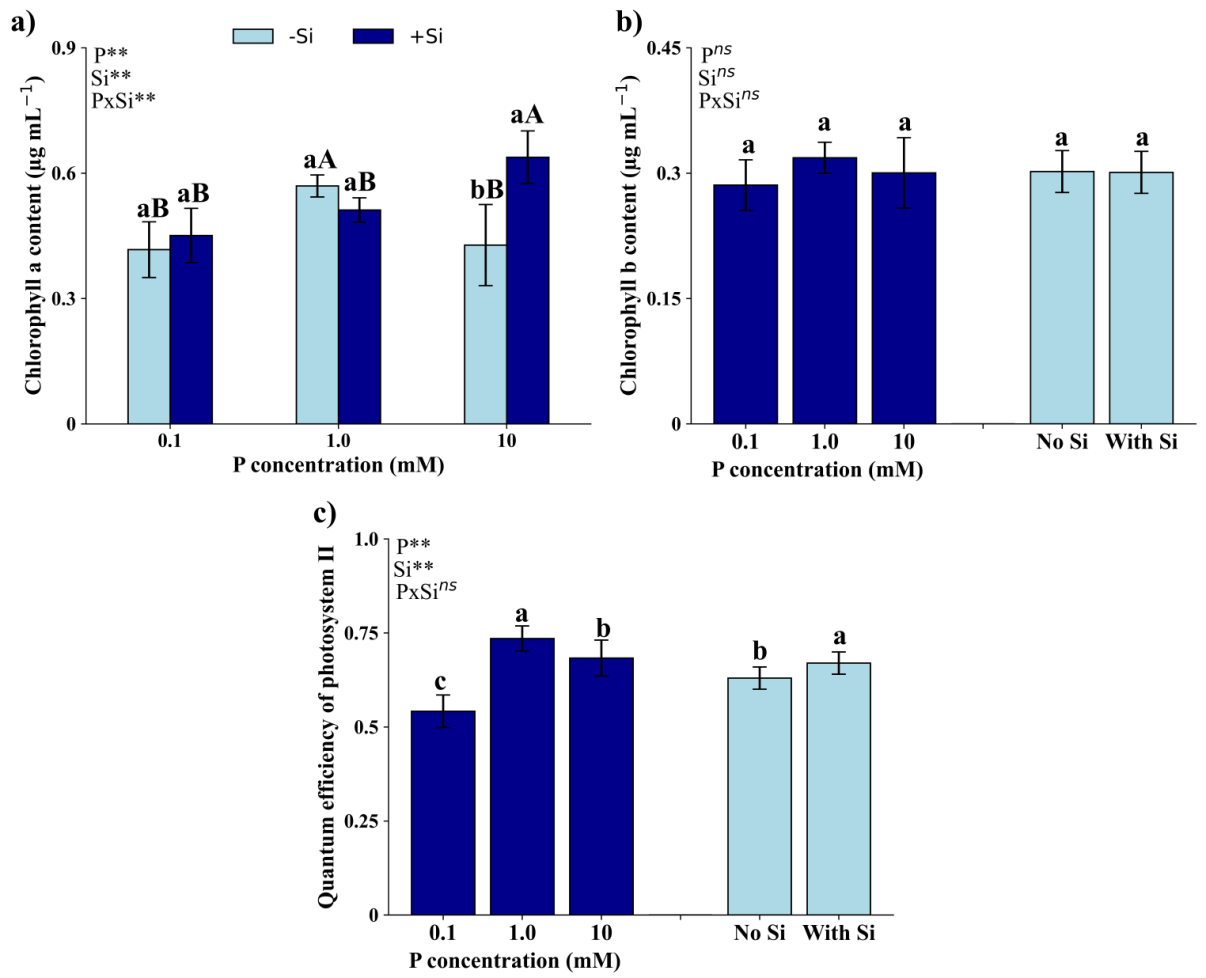
Chlorophyll a content (**a**) and chlorophyll b content (**b**), and quantum efficiency of photosystem II (**c**) of *Eucalyptus grandis* × *Eucalyptus urophylla* seedlings cultivated under different P concentrations (0.1, 1.0, and 10 mM) in the absence and presence of silicon. **, * and ^ns^ – significant at 1 and 5% probability levels and non-significant by the F test. Lowercase letters compare the isolated effects of P or Si in the presence and absence of Si at the same P concentration. Uppercase letters compare different P concentrations under the same Si condition

There was a significant interaction (*p* < 0.01) between P and Si only on chlorophyll *a* content (Fig. 4a). In the absence of Si, the highest chlorophyll *a* content was observed in seedlings under 1.0 mM of P, followed by 0.1 and 10 mM of P. In the presence of Si, the highest chlorophyll *a* content was observed under 10 mM of P, with no difference in seedlings that received 0.1 and 1.0 mM of P. Notably, at a dosage of 10 mM of P, seedlings in the presence of Si showed a higher chlorophyll *a* content, while this variable did not vary in the other dosages studied (0.1 and 1.0 mM) due to the presence of Si.

The chlorophyll *b* content (Fig. 4b) did not differ due to the dosage of P and the presence of Si. On the other hand, quantum efficiency of photosystem II (Fig. 4c) was higher in seedlings that received 1.0 mM of P, followed by seedlings with 10 mM of P and, lastly, by those that received 0.1 mM of P.

### Production of shoot, root, and total dry mass and ratio between root and shoot dry mass

**Fig. 5:**
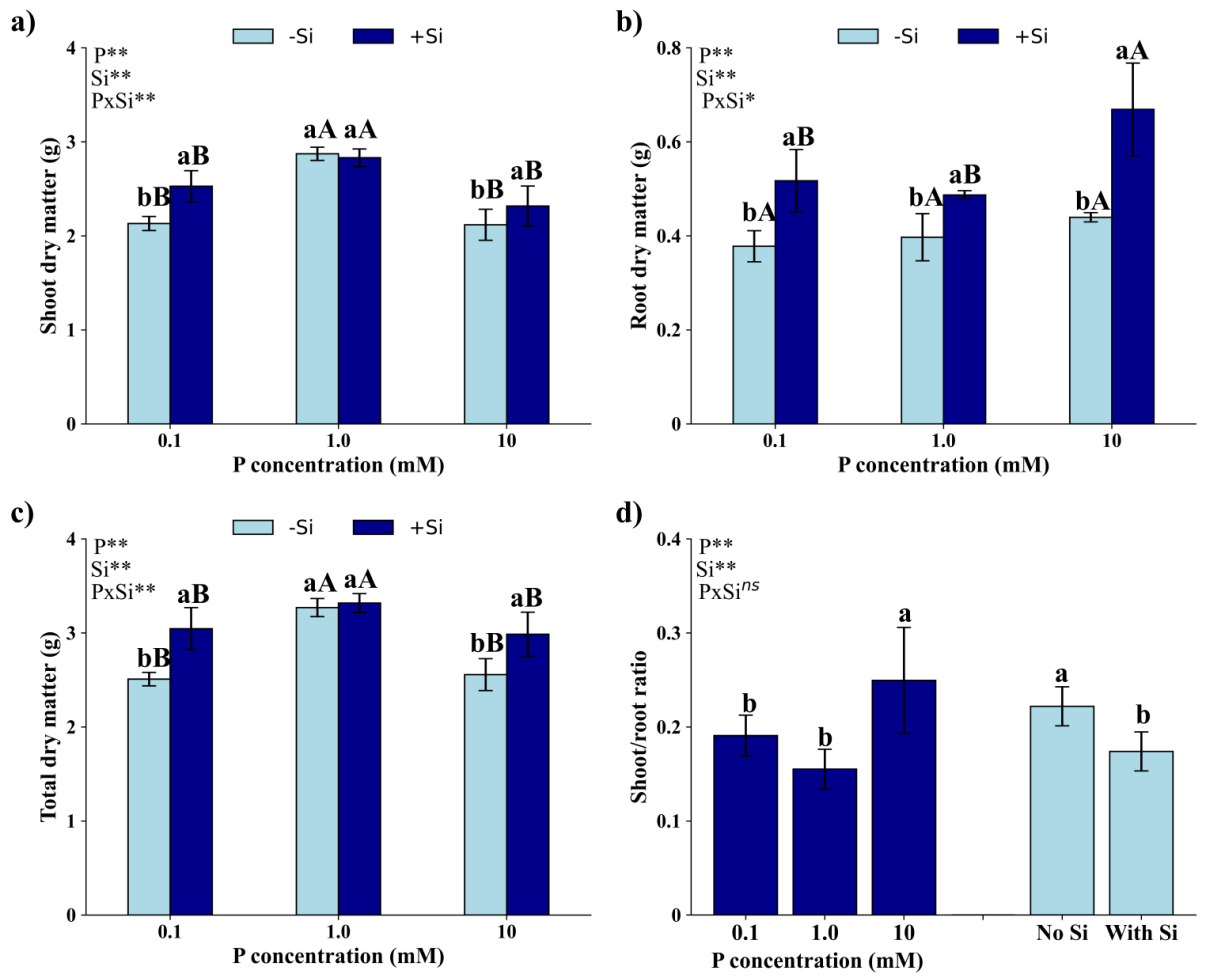
Shoot dry mass (**a**), root dry mass (**b**), total dry mass (**c**), and shoot-to-root dry mass ratio (**d**) of *Eucalyptus grandis* × *Eucalyptus urophylla* seedlings cultivated under different P concentrations (0.1, 1.0, and 10 mM) in the absence and presence of silicon. **, * and ^ns^ – significant at 1 and 5% probability levels and non-significant by the F test. Lowercase letters compare the isolated effects of P or Si in the presence and absence of Si at the same P concentration. Uppercase letters compare different P concentrations under the same Si condition

There was a significant interaction (*p* < 0.05) between P dosages and Si presence or absence on the production of shoot dry mass (SDM) (Fig. 5a), root dry mass (RDM) (Fig. 5b), and total dry mass (TDM) (Fig. 5c), but not on the relationship between SDM and RDM (Fig. 5d).

Production of SDM (Fig. 5a) and TDM (Fig. 5c), regardless of the application or not of Si, was greater under the application of 1.0 mM of P and did not differ in seedlings with 0.1 and 10 mM of P. At concentrations of 0.1 mM and 10 mM of P, seedlings that received Si showed greater production of SDM and total DM, but there was no difference in seedlings grown under 1.0 mM of P due to the presence of Si.

RDM production (Fig. 5b) in seedlings without Si did not differ in relation to P dosages studied. However, in the presence of Si, the highest production of RDM occurred in seedlings grown with 10 mM of P, followed by seedlings with 0.1 mM and 1.0 mM of P, which did not differ from each other. At all P concentrations studied, RDM production increased with the presence of Si.

The ratio between SDM and RDM (Fig. 5d) was higher in seedlings grown with 10 mM of P and the presence of Si reduced the ratio between SDM and RDM.

**Fig. 6:**
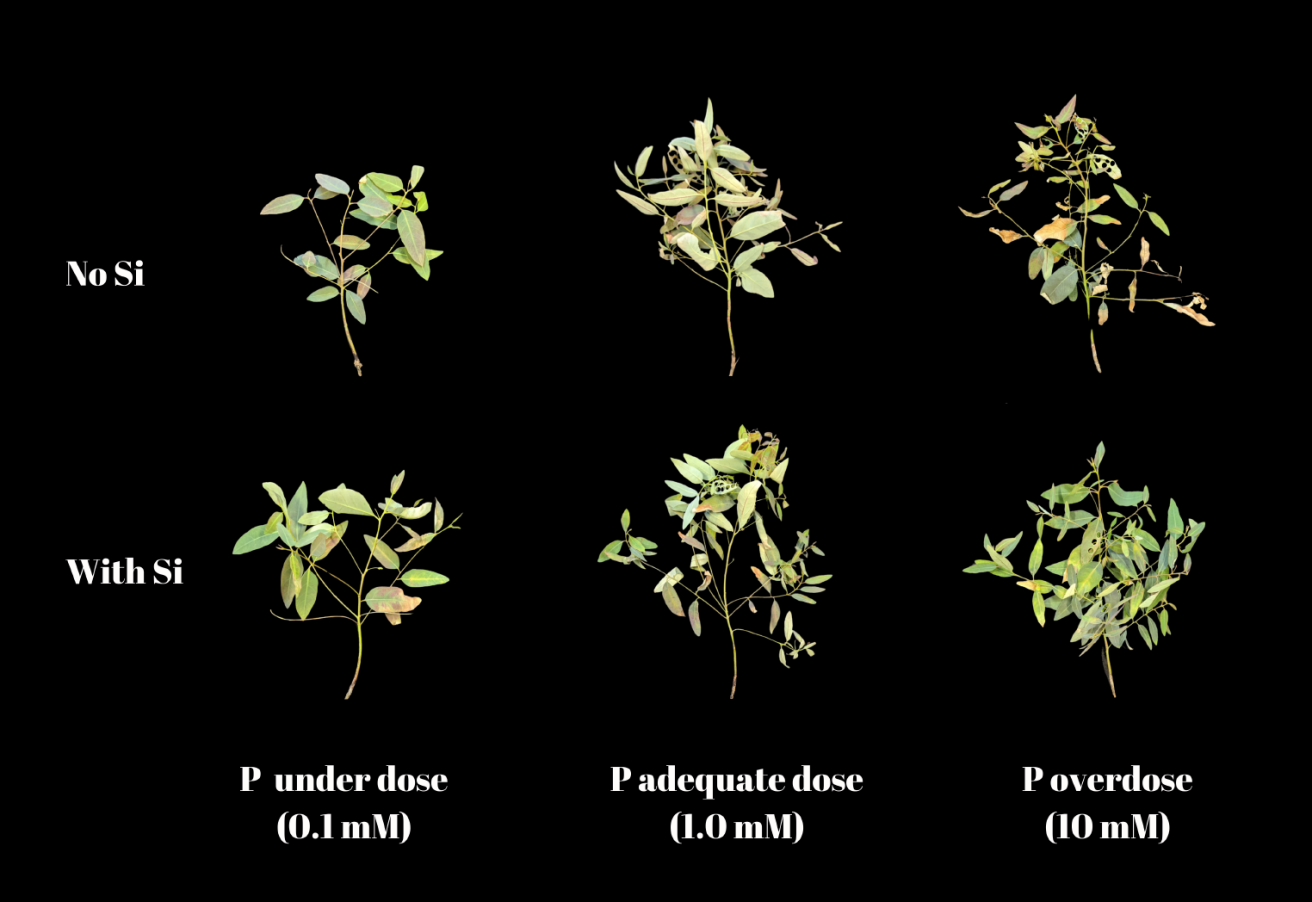
Illustration of the impact of phosphorus (P) concentration and silicon (Si) supplementation on the growth of *Eucalyptus* seedlings. Each row represents seedlings grown under conditions either with or without Si supplementation. The columns correspond to different P treatments: underdose (0,1 mM), adequate dose (1,0 mM), and overdose (10 mM). This comparison underscores that Si supplementation (bottom row) generally enhances plant robustness across all P levels, effectively attenuating the adverse effects observed in seedlings not supplemented with Si (top row)

*Eucalyptus* seedlings without Si supplementation display varying degrees of growth impairments based on the adequacy of P dosage, whether underdose or overdose (Fig. 6). Specifically, seedlings underdose with P exhibit stunted growth, while those overdose with P show a significant decline in health, characterized by a reduced number of leaves. In contrast, seedling with Si supplementation maintain better health and exhibit more robust growth under all P conditions. This indicates that Si has a protective or enhancing effect, helping the *Eucalyptus* better manage P stress.

### Hierarchical data clustering and principal component analyses

**Fig. 7:**
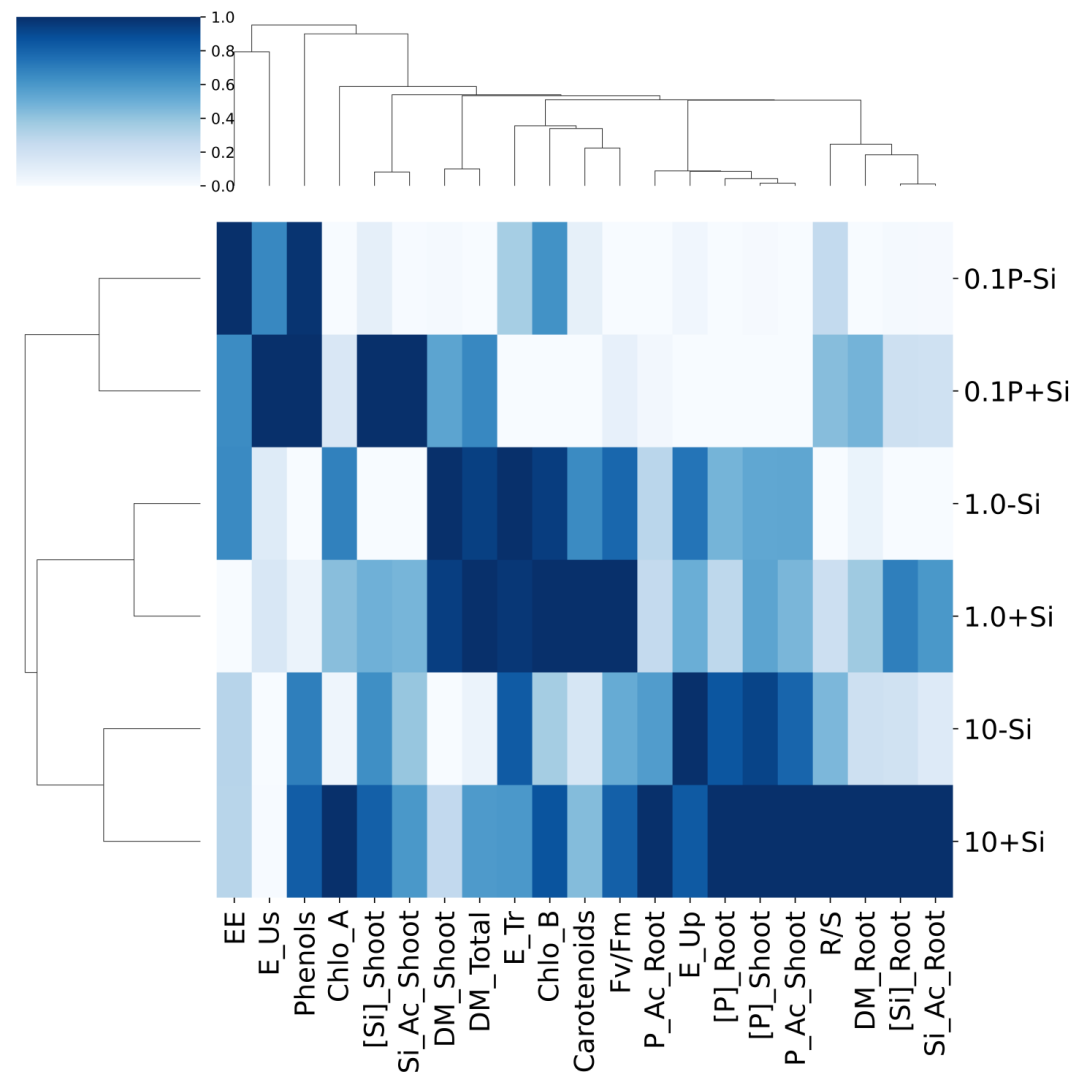
Hierarchical clustering heatmap of response variables of *Eucalyptus grandis* × *Eucalyptus urophylla* seedlings cultivated under different P concentrations (0.1, 1.0, and 10 mM of P) in the presence and absence of silicon (Si). Response variables: Electrolyte leakage (EE), phosphorus use efficiency (E_Us), total phenolic concentration (Phenols), chlorophyll *a* content (Chlo_A), silicon concentration in the shoot ([Si]_Shoot), silicon accumulation in the shoot (Si_Ac_Shoot), shoot dry mass production (DM_Shoot), total dry mass production (DM_Total), transportation efficiency (E_Tr), chlorophyll *b* content (Chlo_B), carotenoids, quantum efficiency of photosystem II (Fv/Fm), phosphorus accumulation in the roots (P_Ac_Root), absorption efficiency (E_Up), phosphorus concentration in the roots ([P]_Root), phosphorus concentration in the shoot ([P]_Shoot), shoot-to-root dry mass ratio (R/S), root dry mass production (DM_Root), silicon content in the roots ([Si]_Root), silicon accumulation in the roots (Si_Ac_Root)

The hierarchical data clustering (HCA) of the data led to the division of treatments into three distinct groups: 0.1 mM of P without Si (0.1P-Si) and 0.1 mM of P with Si (0.1P + Si); 1.0 of P without Si (1.0P-Si) and 1.0 of P with Si (0.1P + Si); and 10 of P without Si (10P-Si) and 10 of P with Si (10P + Si) (Fig. 7). The HCA also indicates less proximity between the 0.1P-Si and 0.1P + Si treatments, followed by the 10P-Si and 10P + Si treatments and, finally, by the 1.0P-Si and 1.0P + Si treatments.

Regarding the 0.1P-Si treatment, an association with electrolyte leakage and phenol production was observed. For the 0.1P + Si treatment, relationships were identified with P use efficiency, content of total phenols, and Si content and accumulation in shoots. The 1.0P-Si and 1.0P + Si treatments demonstrated relationships with SDM production, total DM production, P transport efficiency, and chlorophyll *b* content. The 1.0P + Si treatment also showed an association with the content of carotenoids and quantum efficiency of photosystem II. The 10P-Si treatment was correlated with P absorption efficiency, while the 10P + Si treatment demonstrated a correlation with the chlorophyll *a* content, with the P content and accumulation in roots and shoots, with the mass ratio between SDM and RDM, and with RDM production.

**Fig. 8:**
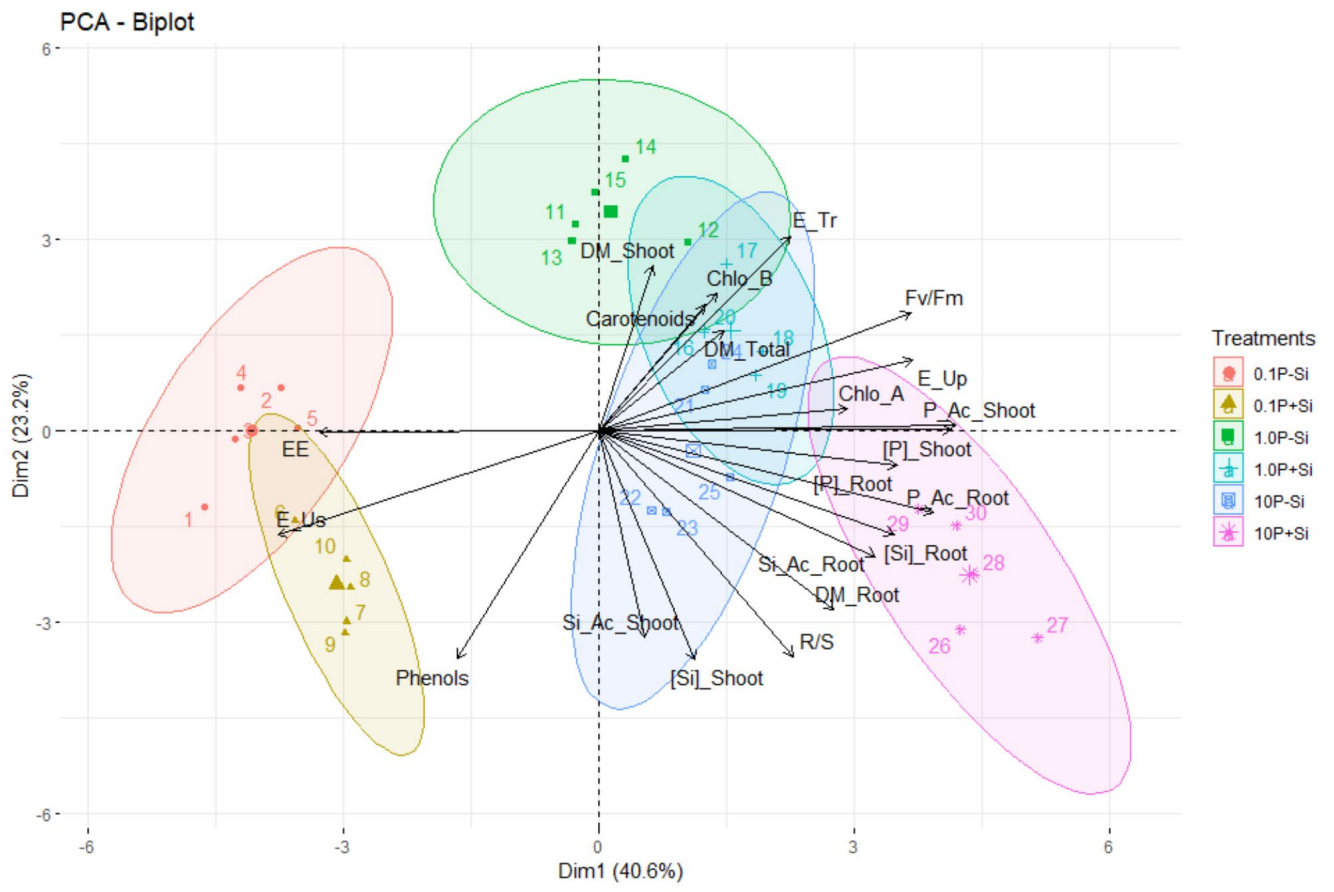
Principal component analysis of response variables of *Eucalyptus grandis* × *Eucalyptus urophylla* seedlings cultivated under different P concentrations (0.1, 1.0, and 10 mM of P) in the presence and absence of silicon (Si). Response variables: Electrolyte leakage (EE), phosphorus use efficiency (E_Us), total phenolic concentration (Phenols), chlorophyll a concentration (Chlo_A), silicon content in the shoot ([Si]_Shoot), silicon accumulation in the shoot (Si_Ac_Shoot), shoot dry mass production (DM_Shoot), total dry mass production (DM_Total), transportation efficiency (E_Tr), chlorophyll b concentration (Chlo_B), carotenoids, quantum efficiency of photosystem II (Fv/Fm), phosphorus accumulation in the roots (P_Ac_Root), absorption efficiency (E_Up), phosphorus concentration in the roots ([P]_Root), phosphorus concentration in the shoot ([P]_Shoot), shoot-to-root dry mass ratio (R/S), root dry mass production (DM_Root), silicon content in the roots ([Si]_Root), silicon accumulation in the roots (Si_Ac_Root)

The PCA elucidated 63.8% of the total variance, with the principal component 1 (PC1) explaining 40.6%, while the principal component 2 (PC2) explained 23.2% (Fig. 8). The following contributed to the explanation of PC1 variance: P concentration and accumulation in shoots and roots, utilization efficiency and absorption of P, quantum efficiency of photosystem II, and electrolyte leakage. In turn, Si concentration and accumulation in shoots, phenol contents, transport efficiency, content of carotenoids, chlorophyll *b* content, the ratio between SDM and RDM, and RDM production contributed to the explanation of PC2 variance. Furthermore, total DM production contributed to explaining the joint variance between PC1 and PC2.

The PCA indicated an association between electrolyte leakage and the 0.1P-Si treatment. Additionally, there was an association between electrolyte leakage, phenol concentration, and P utilization efficiency in the 0.1P + Si treatment. The 1.0P-Si treatment was mainly associated with SDM production, content of carotenoids, and chlorophyll *b* content, while the 1.0P + Si treatment was related to SDM production, content of carotenoids, chlorophyll *a* and *b* content, transport efficiency, quantum efficiency of photosystem II, and total DM production. The 10P-Si treatment was associated with Si concentration and accumulation in shoots, concentration of carotenoids and chlorophyll *a* and *b* contents, P transport efficiency, SDM production, and total DM production. On the other hand, the 10P + Si treatment was associated with the P and Si concentration and accumulation in shoots and roots, the ratio between SDM and RDM, chlorophyll *a* content, P absorption efficiency, and RDM production. These associations provide a more in-depth view of the effects of different treatments on the variables considered, contributing to a more comprehensive understanding of the experiment carried out.

## Discussion

### Phosphorus deficiency and toxicity in seedlings of *Eucalyptus grandis* × *Eucalyptus urophylla*

Studies on P deficiency and toxicity in seedlings of hybrids of *Eucalyptus grandis ***× ***Eucalyptus urophylla* are still incipient in the literature, requiring further research. Plants sensitive to P deficiency normally have inefficient transport systems, while plants sensitive to P toxicity have limited defense mechanisms against nutrient excess [19]. Therefore, it is important to know these aspects of new genetic materials for a more efficient P management of crop in the field.

A study involving 24 different *Eucalyptus* species reported that P accumulation in seedlings varies between 3.8 and 15.9 mg per plant, under conditions of low and adequate P supply, respectively [13]. In our study, the hybrid seedlings of *Eucalyptus grandis *× *Eucalyptus urophylla* showed an accumulation of 1.6, 9.03, and 15.87 mg per seedling with 0.1, 1.0, and 10 mM of P, respectively (Fig. 1). In addition, both seedlings with 0.1 mM of P and with 10 mM of P showed a 15% reduction in total DM, compared to DM of seedlings with 1.0 mM of P, indicating moderate P deficiency and toxicity, respectively [19].

P deficiency can have significant effects on macronutrient nutritional efficiencies in plants. When plants are grown under P deficiency, there is a decrease in physiological characteristics, such as the P content in leaves, growth rate, and the photosynthetic activity [36], also observed in *Eucalyptus grandis *× *Eucalyptus urophylla* seedlings (Figs. 1, 2 and 5). These negative effects of P deficiency can also reduce nutritional efficiencies (Fig. 2).

P also plays an essential role in the synthesis of phospholipids, key elements of cell membranes [19]; thus, P is essential to ensure not only the structural integrity, but also the functionality of the plasma membrane. Plasma membrane stability is compromised under P deficiency, as P in membrane lipids can be remobilized [37] and phospholipids may be replaced by other lipid classes, such as sphingolipids [38], reducing plasma membrane integrity. In seedlings of *Eucalyptus grandis *× *Eucalyptus urophylla*, reduction in plasma membrane integrity due to P deficiency can be observed by the increase in electrolyte leakage (Fig. 3a).

P deficiency can also change the structure of chloroplasts, including a reduction in the number of grana as well as a less organized arrangement [36]. In addition, P deficiency can inactivate photosystem I and suppress the cyclic phosphorylation, reducing quantum yield and efficiency of the electron transport chain. These effects of P deficiency were also observed in seedlings of *Eucalyptus grandis *× *Eucalyptus urophylla* which showed a reduction in chlorophyll *a* content (Fig. 4a) and quantum efficiency of photosystem II (Fig. 4c) under P deficiency.

The effects of P toxicity are rarely reported in the literature and studies on *Eucalyptus grandis *× *Eucalyptus urophylla* seedlings are incipient. However, P toxicity is known to inhibit the content of photosynthetic pigments [39], the net rate of photosynthesis [40] and, consequently, dry matter production of plants [41]. These results were also observed in the present study, as the hybrid seedlings showed a reduction in the chlorophyll *a* content (Fig. 4a), quantum efficiency of photosystem II (Fig. 4c), and TDM production (Fig. 5).

These results allow to accept the first hypothesis that *Eucalyptus grandis *× *Eucalyptus urophylla* seedlings are sensitive to P deficiency and toxicity, leading to a reduction in nutritional efficiencies, an increase in electrolyte leakage, a reduction in pigment concentration and quantum efficiency of photosystem II and, consequently, a reduction in dry matter production of seedlings.

### Silicon mitigating P deficiency and toxicity in seedlings of *Eucalyptus grandis* × *Eucalyptus urophylla*

*The Eucalyptus* species presents low concentrations of Si; however, our results indicate considerable Si concentrations in roots (Fig. 1f). This result is expected, because as *Eucalyptus* belongs to the Myrtaceae family, it is classified as a non-Si-accumulating plant [20]. These plants have the capacity to absorb Si in the soil solution in the form of monosilicic acid [42], but they have a low capacity to transport Si to shoots, generating a high Si concentration in roots. Seedlings of *Eucalyptus grandis *× *Eucalyptus urophylla* showed Si concentration of 9.91 mg kg^− 1^ in roots of plants cultivated with Si, 369% higher than the concentration in shoots of the same plants, which was 2.11 mg kg^− 1^ (Fig. 1) The higher Si concentration in roots of seedlings increases RDM production (Fig. 8), evidenced by the 40% increase in RDM production (0.56 g) in the presence of Si, compared to seedlings cultivated in the absence of Si (0.40 g) (Fig. 5).

The higher Si concentration in roots may be associated to greater efficiency of P absorption due to the Si effect on the positive regulation of the expression of transporter genes responsible for the uptake of phosphate ions [43]. In addition, Si can optimize nutrient transport efficiency throughout the plant, promoting a wider distribution of essential elements [44]. Si is also related to the stoichiometric replacement of carbon (C), resulting in metabolic energy savings for the plant, which can be used to synthesize other non-structural organic compounds, consequently increasing the efficiency of nutritional use [45, 46]. This effect of Si has been widely reported in several crops grown in nutrient solutions [47–50]. However, in *Eucalyptus grandis *× *Eucalyptus urophylla* seedlings, the effect of Si on nutritional efficiencies is still incipient in the literature.

The results of this work show that Si reduces absorption efficiency, does not affect transport efficiency, and increases P use efficiency in plants grown with 0.1 mM of P (Fig. 2c). The negative effect of Si on absorption efficiency may be related to its action to increase RDM production (Fig. 5b), a variable that is inversely proportional to absorption efficiency [32]. The greater P utilization efficiency in seedlings grown with 0.1 mM of P in the presence of Si can be attributed to the stoichiometric effect of Si [46, 49–52], an aspect that requires additional studies to evaluate this effect in *Eucalyptus grandis *× *Eucalyptus urophylla* seedlings. However, as reported in this study, the beneficial effect of Si to reduce oxidative stress and increase non-enzymatic antioxidant mechanisms (Fig. 3), as well as increase the chlorophyll *a* content and quantum efficiency of photosystem II (Fig. 4c), may have contributed to augmenting utilization efficiency of P by seedlings grown with 0.1 mM of P. The multivariate analyses of hierarchical clustering (Fig. 7) and principal components (Fig. 8) showed a high association between the presence of Si and efficiencies of absorption, transport, and utilization in seedlings grown with 10, 1.0, and 0.1 mM of P, respectively. These results indicate a correlation between the presence of Si and nutritional efficiencies in *Eucalyptus grandis *× *Eucalyptus urophylla* seedlings compared to the absence of Si.

Another beneficial effect of Si in attenuating P deficiency is evidenced by the reduction in electrolyte leakage (Fig. 3a) and the increase in the total phenol contents (Fig. 3b). Additionally, electrolyte leakage is associated to seedlings with 0.1 mM of P, both in the presence and absence of Si (Fig. 7). However, seedlings with 0.1 mM of P in the presence of Si demonstrate a high association with the phenol contents. Phenolic compounds act as neutralizers of reactive oxygen species (ROS), neutralizing and preventing oxidative damage [53–55]. The multiple hydroxyls and carboxylic groups in phenolic compounds help to form stable complexes with proteins, inhibiting the development of free radicals [56, 57]. .

*Eucalyptus grandis *× *Eucalyptus urophylla* seedlings grown with 10 mM of P did not show an increase in electrolyte leakage (Fig. 3a). On the other hand, when in the presence of Si, the seedlings showed a higher chlorophyll *a* content (Fig. 4a) and greater quantum efficiency of photosystem II (Fig. 4c), when compared to plants grown with 0.1 mM of P. Seedlings grown with 10 mM of P in the presence of Si showed a high association with the chlorophyll *a* content and the quantum efficiency of photosystem II (Fig. 8). Si can directly and indirectly favor photosynthetic reaction centers [58]. Si favors greater cell wall rigidity, forming a double layer of Si in the leaf epidermis thus improving leaf architecture and light absorption capacity, resulting in less energy loss due to fluorescence [59] and consequently greater efficiency of quantum photosystem II, as previously reported in *Eucalyptus* spp [21]. .

In summary, our results demonstrate the beneficial influence of Si with improvements in nutritional efficiency, oxidative metabolism, and photosynthetic parameters, boosting the development of *Eucalyptus grandis *× *Eucalyptus urophylla* seedlings, observed by the greater SDM, RDM, and TDM production. These results allow to accept the second hypothesis of this work, in which the supply of Si is capable of attenuating the negative effect of P nutritional disorder through physiological and nutritional mechanisms and by reducing the oxidative stress and increasing photosynthetic parameters and nutritional efficiencies.

The results of this research propose a sustainable strategy based on the use of Si and have global implications given the fact that *Eucalyptus* cultivation has expanded worldwide, mainly in regions with P-deficient soils. Future studies should investigate new hybrids of the *Eucalyptus* species, mainly in the field of ​​molecular biology, to better understand whether the genes activated by Si are responsible for beneficial physiological and nutritional changes in plants cultivated under P nutritional disorder. This molecular approach is essential for deciphering the specific genetic pathways influenced by Si, providing a clearer picture of its impact at a cellular level. Additionally, it is crucial to explore the effects of Si on various enzymatic and non-enzymatic antioxidant defense systems in *Eucalyptus* seedlings to comprehensively understand its role under such stress conditions.

## Conclusion

The results of this study indicate that *Eucalyptus grandis *× *Eucalyptus urophylla* seedlings are sensitive to P deficiency and toxicity and Si has shown a beneficial effect, attenuating P nutritional disorder by reducing the oxidative stress, and favoring the non-enzymatic antioxidant system and photosynthetic parameters.

## Data Availability

The datasets generated and/or analyzed during the current study are available from the corresponding author on reasonable request.
